# LV reverse remodeling imparted by aortic valve replacement for severe aortic stenosis; is it durable? A cardiovascular MRI study *sponsored by the American Heart Association*

**DOI:** 10.1186/1749-8090-6-53

**Published:** 2011-04-14

**Authors:** Robert WW Biederman, James A Magovern, Saundra B Grant, Ronald B Williams, June A Yamrozik, Diane A Vido, Vikas K Rathi, Geetha Rayarao, Ketheswaram Caruppannan, Mark Doyle

**Affiliations:** 1 Center for Cardiovascular Magnetic Resonance Imaging, The Gerald McGinnis Cardiovascular Institute, Department of Medicine, Division of Cardiology, Allegheny General Hospital, Drexel University College of Medicine, Pittsburgh, Pennsylvania, USA; 2 Division of Internal Medicine, Allegheny General Hospital, Pittsburgh, Pennsylvania, USA; 3 Department of Surgery, Division of Cardiothoracic Surgery, Allegheny General Hospital, Pittsburgh, Pennsylvania, USA

## Abstract

**Background:**

In patients with severe aortic stenosis (AS), long-term data tracking surgically induced effects of afterload reduction on reverse LV remodeling are not available. Echocardiographic data is available short term, but in limited fashion beyond one year. Cardiovascular MRI (CMR) offers the ability to serially track changes in LV metrics with small numbers due to its inherent high spatial resolution and low variability.

**Hypothesis:**

We hypothesize that changes in LV structure and function following aortic valve replacement (AVR) are detectable by CMR and once triggered by AVR, continue for an extended period.

**Methods:**

Tweny-four patients of which ten (67 ± 12 years, 6 female) with severe, but compensated AS underwent CMR pre-AVR, 6 months, 1 year and up to 4 years post-AVR. 3D LV mass index, volumetrics, LV geometry, and EF were measured.

**Results:**

All patients survived AVR and underwent CMR 4 serial CMR's. LVMI markedly decreased by 6 months (157 ± 42 to 134 ± 32 g/m^**2**^, p < 0.005) and continued trending downwards through 4 years (127 ± 32 g/m^**2**^). Similarly, EF increased pre to post-AVR (55 ± 22 to 65 ± 11%,(p < 0.05)) and continued trending upwards, remaining stable through years 1-4 (66 ± 11 vs. 65 ± 9%). LVEDVI, initially high pre-AVR, decreased post-AVR (83 ± 30 to 68 ± 11 ml/m2, p < 0.05) trending even lower by year 4 (66 ± 10 ml/m^**2**^). LV stroke volume increased rapidly from pre to post-AVR (40 ± 11 to 44 ± 7 ml, p < 0.05) continuing to increase non-significantly through 4 years (49 ± 14 ml) with these LV metrics paralleling improvements in NYHA. However, LVmass/volume, a 3D measure of LV geometry, remained unchanged over 4 years.

**Conclusion:**

After initial beneficial effects imparted by AVR in severe AS patients, there are, as expected, marked improvements in LV reverse remodeling. Via CMR, surgically induced benefits to LV structure and function are durable and, unexpectedly express continued, albeit markedly incomplete improvement through 4 years post-AVR concordant with sustained improved clinical status. This supports down-regulation of both mRNA and MMP activity acutely with robust suppression long term.

## Introduction

In patients with severe aortic stenosis (AS), compensatory left ventricular hypertrophy (LVH) is the predominate mechanism manifest to attempt to normalize the markedly elevated afterload imposed at the aortic valve level [1]. Overtime this initially beneficial response leads to deleterious downstream effects not limited to mismatched neovascularization relative to the extent of left ventricular (LV) hypertrophy, supranormal LV performance likely due to geometic remodeling and marked interstial fibrosis due to collagen deposition that eventually leads to codominant explanations for the often pronounced hypertrophy often seen in late stage AS [2-5]. It is for these reasons that the goal of aortic valve replacement (AVR) is aimed. AVR is designed to relieve valvular afterload but with the cardinal physiologic effect directed at inducing regression of the excessive LVH. In this manner it has long been known that there is a survival advantage in those who receive AVR as compared to those who, for other reasons, fail to undergo corrective surgery. However, the long-term data tracking the surgically induced beneficial effects of afterload reduction on reverse LV remodeling are available only in limited fashion. Moreover, the majority of the available data exists in echocardiographic literature, is pertinent to remodeling concepts is available short term [6,7], but only in limited fashion beyond one year [8-12].

Cardiac magnetic resonance imaging (CMR) is the 'gold standard' for measuring cardiac volumetrics LV mass and offers the ability to track changes in LV metrics with innordinantly small numbers due to its inherent high spatial resolution and low intraobserver variability [13]. Indeed, as compared to echocardiography, Bottini *et al *demonstrated that if one wished to be able to detect a 10 gram regression in LV mass with an alpha of 0.05 and a beta of 0.80 it would require 550 patients, whereas *only *17 patients were necessary by CMR [14]. This represents over a log-fold reduction in the number of patients required in order to detect a beneficial effect by CMR over the more commonly used modality, echocardiography. Thus, the pattern and temporal manner in which LVH regresses, currently unknown, conceivably should be discernable over a long period of time pre and post-AVR non-invasively via CMR in a small number of patients providing answers as to the completeness and durability of LVH regression following AVR.

## Hypothesis

We hypothesize that progressive LV reverse remodeling changes following AVR are detectable by CMR and changes in LV structure and function, once triggered by AVR, continue for an extended period.

## Methods

### Population

Patients referred for AVR were enrolled after institutional review board (IRB) approval and signed consent obtained. All patients were identified via standard clinical metrics independent of CMR evaluation chiefly through cardiac catheterization and/or echocardiography. To provide homogeneity in the pathology of AS, patients were excluded if there was aortic or mitral regurgitation assessed by echocardiographic imaging as greater than moderate (>2+), mitral stenosis, prior valve replacement, myocardial infarction, history of hypertension, coronary artery bypass grafting (CABG) or angioplasty. Specific contraindications to CMR were presence of a pacemaker, defibrillator, history of metal fragments, implants, cerebrovascular clips or claustrophobia.

### CMR Imaging

The 3D CMR methodology has been described elsewhere [15,16]. Briefly, using a General Electric (Milwaukee, Wisconsin) 1.5T Excite EKG-triggered CMR system (50 mT/m maximum gradient strength, 150 mT/m/ms maximum slew rate), scout images were obtained to plan double-oblique views in horizontal and vertical long-axis views from which short-axis contiguous 8 mm slices traversing the mitral valve plane through LV apex were acquired using a steady-state free precession (FIESTA) cine sequence with a field of view 38 cm^**2**^, matrix 256 × 192, flip angle 45°. The temporal resolution was 30 ± 3 ms,100% phase FOV and 0.75 NEX, TR 3.2 ms and TE 1.4 ms. From the short-axis images, LV end-diastolic volume (LVEDV), LV end-systolic volume (LVESV), LV stroke volume (LVSV), LV ejection fraction (EF), and LV mass were measured and indexed to BSA. LV mass was derived via Simpson's method multiplied by the specific gravity of myocardium (1.055 g/ml). Image acquisition was kept constant to include LV basal plane-registration throughout the study and between patients to minimize variability in measurements.

Phase velocity mapping (PVM) was employed to quantitate 3D peak and mean aortic transvalvular gradients in the through and in-plane slices. Velocity encoding was set at 350-550 cm/sec with encoding in the x, y and z directions. PVM was resolved into 60 phases/cardiac cycle achieving high temporal resolution(19 ± 3 ms). ROI's were manually drawn encircling the entire supravalvular plane for complete interrogation of all velocities, as opposed to the 'ice-pick' view employed by echocardiography. 2D transthoracic and/or transesophageal echocardiography was also performed for independent clinical assessment of AS.

**Figure 1:**
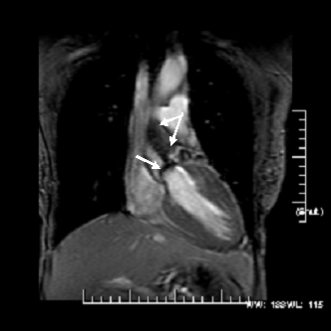
**A coronal view from a steady-state free precession acquisition demonstrating the heavily calcified (arrow) and restricted aortic valve leaflets with a intervoxel dephasing defect as depicted by the systolic turbulence (bifid arrow) radiating into the proximal ascending aorta**. In itself, this is indictative of a highly velocity jet consistant with severe AS. Using phase velocity mapping to formally quantitate the mean and peak transvalvular gradients, they were 53 and 78 mmHg, respectively; severe AS.

All images were analyzed offline on semi-automatic MASS Plus and Flow programs (Medis, The Netherlands). CMR imaging was performed (5 ± 3 days) prior to AVR, 6 month and 1 year and up to 4 years post-AVR. An independent comparison of AS degree assessed by each modality (CMR and echocardiography) was performed for future reference and was recorded, see image (Figure 1). All data was analyzed by a single dedicated CMR technologist (JAY or RW) throughout the study period to minimize interobserver variability with all images blindly over-read by a dedicated cardiologist (RWWB or VR). The mean imaging time for the patients was 54 ± 15 minutes.

Mitral regurgitation was retrospectively semiquantitativly assesed as a function of the intervoxel dephasing artifact from the vertical and horizontal long-axis using the steady state free-precession (FIESTA) dynamic cine sequence at each time point. Measurements of the mitral annulus, valve tenting angle and valve tenting area were meaured using standard approaches in 2D from the vertical and horizontal long-axis.

### Statistics

Continuous variables were reported as mean ± 1 SD. Categorical variables were reported as percentages with 95 percent confidence intervals. Serial comparisons pre- to post-AVR were performed by the paired t-test. Effects across groups were analyzed using one-way analysis of variance (ANOVA) and repeated-measures ANOVA was performed for comparisons over time. Statistical analyses were performed using SPSS for Windows, version 11.0 (SPSS, Inc., Chicago). All statistical comparisons were performed using two-tailed significance tests with a 'p' value of < 0.05 considered statistically significant.

## Results

Twenty-four patients underwent pre-AVR CMR. A random subset of patients who were imaged at the 6 month and 1 year time point were specifically invited back to be imaged at a fourth very late time point and underwent post-AVR imaging at 6 ± 2mo and 1 yr ± 2mo and up to 4 years (one patient imaged at 3.5 years) for 40 total time points. Thus, ten patients (67 ± 12 years, 6 female) with severe, but reasonably well compensated AS, underwent CMR pre-AVR and 3 subsequent time points post-AVR. Two patients were classified as NYHA class III, all others were < NYHA II. Four patients had concomitant CAD but were without significant differences in their peak and mean transvalvular gradient by either echocardiography or CMR. There was no significant difference between the CMR derived mean and peak transvalvular gradients (47 ± 12 and 70 ± 24 mmHg, respectivly) vs. the mean and peak gradients as measured by echocardiography (42 ± 10 and 68 ± 21, respectively) though CMR velocities tended to be higher, p=NS). Stated alternatively, there was no difference in the number of patients with >4 m/s peak transvalvular gradient as measured by CMR and echocardiography (7 vs. 7 patients). The mean NYHA pre-AVR was 2.5 ± 1.2.

**Figure 2:**
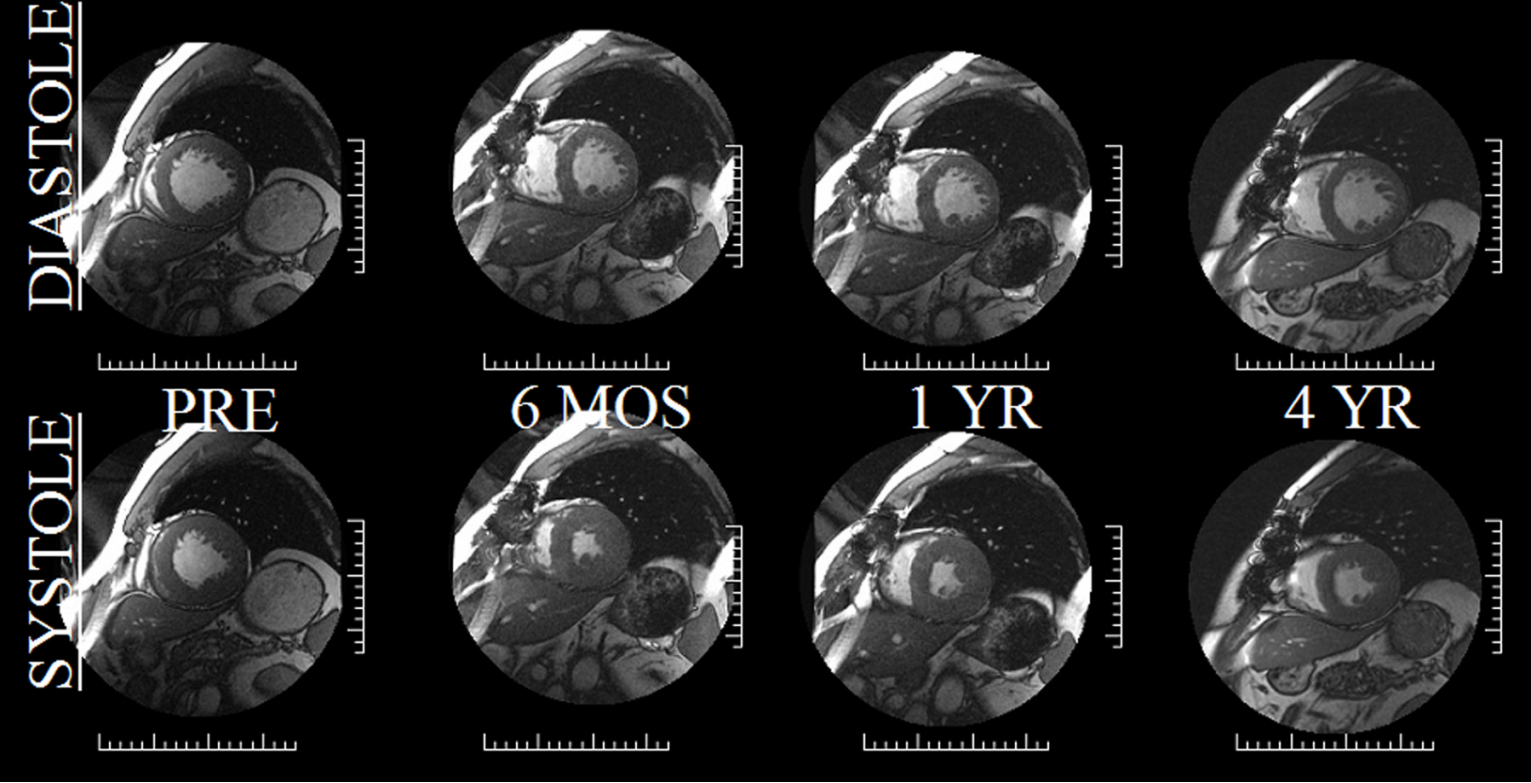
**Serial cardiovascular MRI mid short-axis images in diastole (top row) and systole (bottom row) in a 76 WM taken the day prior to AVR, 6 months, one year and 4 years following AVR**. The LV mass decreased from 186 to 154 g over the first 6 months to only regress to 132 g over the next 3 1/2 years demonstrating the early-rapid and late-slow pattern of LVH regression. Similarly, LVEF markedly improved after afterload relief from 54% to 60% in the first 6 months with no further improvements over the ensuing 3 1/2 years (62%).

**Figure 3:**
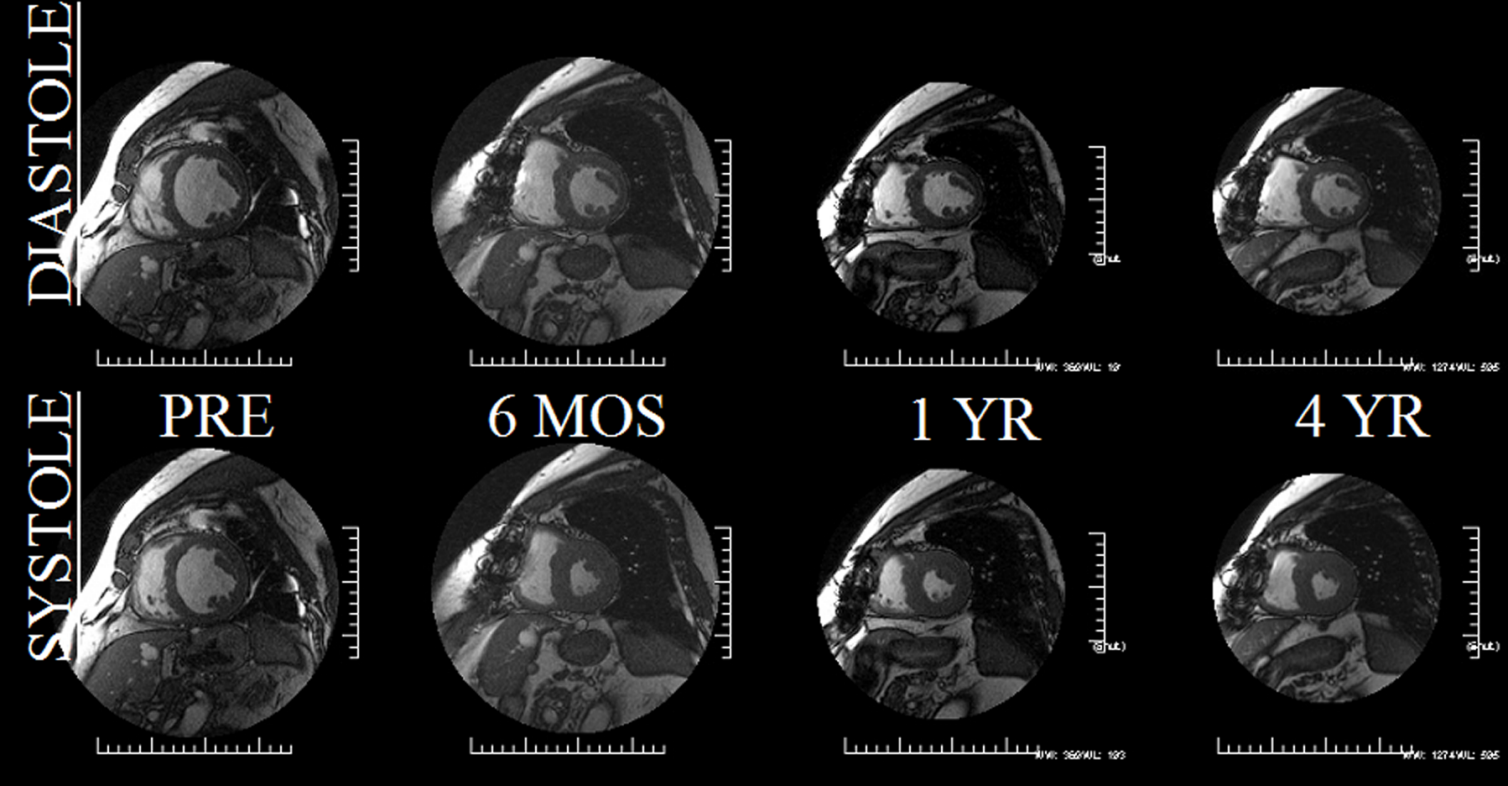
**Demonstrating that, despite marked afterload mismatch in a 55YOWM with an LVEF 23% and LV mass of 251 g, surgical relief of afterload in a patient with demonstrated myocardial reserve (mean/peak gradients of 52 and 33 mmHg, respectively) can ensue with striking improvements in LVEF and LV mass (57%EF and 197 g at 6 months post-AVR) with minimal change by year 4 (LVEF 56% and LV mass 158 g)**. The initial improvements in morphometrics and volumetrics paralled marked improvements in the patients clinical response, again most evident within the first 6 months post-AVR.

All patients had severe LVH prior to undergoing AVR. Following AVR, LVMI markedly decreased at 6 months (157 ± 42 to 134 ± 32 g/m^2^, p < 0.005) and continued to further trend downward at 4 years (127 ± 32 g/m^2^; p = NS), see Figures 2 and 3. Similarly, EF increased pre to post AVR (55 ± 22 to 65 ± 11%, (p < 0.05)) and continued trending upward, however remaining statistically stable at years 1-4 (66 ± 11 vs. 65 ± 9%). LVEDV index, initially high pre-AVR, declined post-AVR (83 ± 30 to68 ± 11 ml/m^2^, p < 0.05) trending even lower by year 4 but again remaining statistically insignificant (66 ± 10 ml/m^2^).

LV stroke volume index increased rapidly from pre to post-AVR (40 ± 11 to 44 ± 7 ml/m^2^, p < 0.05) trending to increase at 4 years (49 ± 14 ml/m^2^) but also remaining statistically insignificant as compared to the 6 month time period.

**Figure 4:**
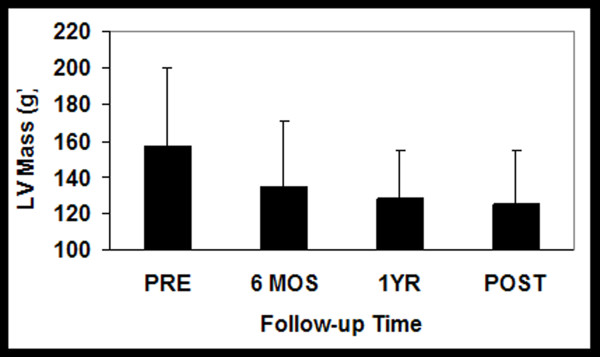
**Plots of the temporal nature of the pattern of LVH regression serially out to 4 years**. Note the immediate LVH regression sparked by the massive afterload relief by AVR. However, the trajectory of initial regression at 6 months would have predicted a far greater mass reduction then evident at 4 years.

However, despite the relatively long term follow-up there remained incomplete LV mass regression, failing to return to historic age-matched control level (59 ± 11 g/m^2^) [17], see Figure 4. Likewise, LVEDVI did not normalize, remaining above historic age-matched controls [17].

The 3D CMR equivalent to echocardiographic *relative wall thickness *(RWT), an indicator of 1D LV geometry, is the *mass/volume ratio*. As a 3D metric, the mass/volume ratio has obvious advantages over any 1D measurement and accordingly is used to more definitively relate changes in LV geometry over time. The mass/volume ratio demonstrated no change initially (1.9 to 2.0 at 6 months) remaining unchanged at 1 year (2.0) and out to 4 years (1.9), p = NS between all.

While all metrics except for EF were markedly elevated as compared to normals, despite substantial metric approaching within 2 standards deviations of normal. LV mass index specifically remained >5 standard deviations above normal.

The temporal pattern for regression for all stand-alone metrics including EF demonstrated that a minimum of nearly 50% of the change that was to be evident by 4 years occurred *within *the first 6 months. For instance, for LVMI, 76% of the mass that regressed by year 4 did so in the first 6 months while for LVEDV, 88% of the reduction occurred within the first 6 months. Likewise, nearly all (91%) of the final EF achieved was present within the first 6 months with no significant changes apparent afterwards. Due to the near parallel changes in LVMI and LVEDVI, by definition, there would be no discernable temporal pattern in the mass/volume ratio over the entire 4 years.

### Mitral Regurgitation

**Figure 5:**
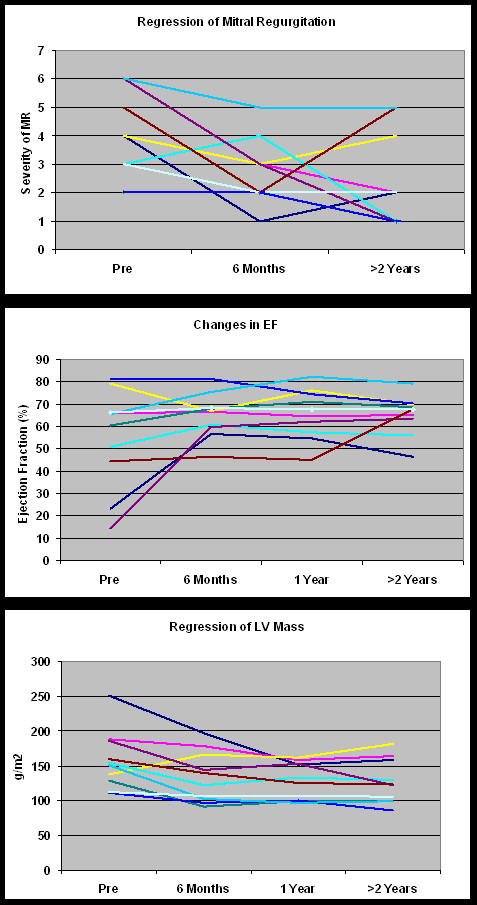
**(Fig A, B, C) Change in mitral regurgitation that ensues upon the relief of afterload by AVR**. All but 2 patients had CMR defineable reduction in their MR grade (defined herein as 0 through 7 representing no (absent) through 2+ (moderate) MR. In those 2 patients the least amount of LV remodeling was present suggesting that effective mass/volume normalization is an important mechanism towards stabilizng and eventual MR relief as it is in its initiating pathophysiology. (Note, superimposition prevents all 10 patients from being displayed).

It should be noted that the primary objective of the study was to interrogate a pure human pressure afterload model of AS induced concentric LVH pathology, such that any significant amount of potential eccentric LVH due to volume overload was *a priori *excluded. Nevertheless, a biologic signal to assess whether the degree of mitral regurgitation (MR) could be favorably influenced might be deducible from this population. Pre-AVR, the grade of MR was '0' through '2+' (moderate MR). Post-AVR the MR remained stable or decreased late in 80% and increased in two patients (0-trace in one patient and trace to 2+ another patient (both in patients who had the least amount of reverse remodeling), see Figure 5. The favorable changes in LV mass and LV EDVI post-AVR were highly correlated with MR improvement (r = 0.51 and 0.60, respectively). While EF increased, it was not well correlated (r = 0.31) with MR reduction post-AVR, while LV sphericity (r/h) just failed to reach statistical significance with the improvement in mitral regurgitation.

### Clinical Sequelae

Paralleling improvements in CMR derived LV volumetrics and morphometrics including mitral regurgitation, there were concordant improvements in NYHA class. Pre-AVR NYHA was 2.5 ± 1.2 and rapidly improved to 1.6 ± 0.9 at 6 months and 1.6 ± 0.9 at 1 year but remained statistically insignificantly improved out to 4 years as compared to the interim time points (1.4 ± 1.1). However, as compared to pre-AVR, there was an important significant difference over time by 4 years (p < 0.05).

## Discussion

Due to excessive afterload imposed on the LV from the markedly restricted valvular narrowing in patients with severe but compensated AS, substantial LVH is typically apparent. While initially a favorable compensatory response to the often extraordinary intraventricular pressure, left unchecked, LVH heralds a slow inexorable deterioration in cardiac function promulgated by further changes at the myocardial and interstitial level. To the extent that these now pathologic process are reversible is unclear. To be sure, it is well known that the epidemiological post-surgical effect is extremely favorable nearly restoring survival by actuarials back to the pre-morbid state. However, the nature, extent and temporal pattern of these surgically induced reverse remodeling effects are much less clear. Limited attempts to track LVH regression after AVR have been performed by 2D echocardiography but generally over short periods of time, often under one year post-AVR. To our knowledge this is the first attempt to apply the long known reference standard CMR, interrogating LV volume, EF and LV mass, incorporating long-term remodeling to this issue.

### CMR

CMR has an ability to detect exceedingly small aliquots of myocardial mass change (intraobserver variability of 2.5 g) while detecting changes in volumetrics such that EF changes of 1.5%, while at lower limits of intraobserver variability, are discernable and relevant. This provides for an unparalleled ability for CMR to be used to interrogate pre and post-AVR changes in a reliable and clinically relevant manner. As described above, CMR retains the ability to discriminate such findings in historically smaller populations then previously considered via other modalities due to its ultra high spatial resolution often leading to log-fold less patient requirements to achieve statistical significance yet retaining preserved power^14^.

### LV Metrics after AVR

In this study, after the initial beneficial effects imparted by afterload relief by AVR in severe AS patients, there are as expected, marked improvements in LV reverse remodeling. We have shown, via CMR, that surgically induced benefits to LV structure and function, including favorable alterations in LV geometry, are definable, durable *and*, unexpectedly, show continued improvement up to 4 years concordant with sustained improvement in clinical status. That these finding have awaited recognition and substantiation for decades detracts nothing from the expected, even predicatable reasoning that they *would *be present since there is a clear survival advantage for those that do undergo AVR as compared to those that choose not to, (depite being equivalent in all other demographic and pathological characteristics).

However, the observed pattern of reverse remodeling has never been defined before in this patient population and was unexpected in its temporal trajectory. Fully 75% of the LV mass regression that was to occur did so within the first 6 months following AVR. In fact, nearly 90% of the change in volumetrics (LVEDVI and LVEF) were completed in the first 6 months with clinically insignifcant changes detected subsequently. In that the first oportunity to detect the changes was by protocol defined at 6 months, it is conceivable that one or more of these metrics had their improvement at an even earlier time course.

### Incomplete LVH Regression after AVR

The most striking finding in this study was not the extent of LV reverse remodeling that was found but that, despite serial follow-up up to 4 years, there is a distinct failure to normalize LV mass. LV mass remained >5 standard deviations above normal for >85% of the population without explanations on the basis of age, sex, CAD, and pre-AVR metrics such as gradient, valve type, cross-clamp time via multivariate analysis as they were unable to account for the failure of LVH regression. Should this be surprising to us? Are there inferences in the literature that might guide us to this conclusion? Several avenues of support for this finding are available as well as some that require a more considered approach.

First off, AVR itself does not restore the transvalvular gradient to normal. Despite the advent of increasingly lower profile aortic valves, to include the Toronto SPV (used in 40% of this patient group), residual gradients exist and to the extent that they remain, invariably contribute to residual afterload and obligatorily thwart complete LV mass regression. In most cases, however, the ratio of residual to initial gradient is likely to be low ( < 20%) thereby having only modest impairment of eventual LV mass regression.

Secondly, at the same time the afterload is surgically relieved at the valve level, *supravalvular *afterload is likely to be increasing due to aortic and peripheral changes in compliance and arterial inelasticity due to aging. The surgically induced relief of afterload may be counterbalanced by the resultant increase another type of afterload; arterial hypertension [18].

Another mechanism thwarting regression of LVH is less obvious. Classically, the hypertrophic process is thought to be composed chiefly of sarcomeres being laid down in parallel resulting in concentric hypertrophy. This process is governed mostly by mRNA expression. Naturally, LVH regression therefore would be thought as a reversal of this process following AVR. What has become clear however is that the pathologic perturbation in AS is not confined at the ventricular level only to the myocyte [19]. The extracellular matrix, primarily composed of collagen deposition as a response to the pressure overload and probably due to increased perimysial fibers to translate the generated myocardial deformation, expands to become a very significant proportion of the total LV mass [20,21]. Its regulation and subsequent regression is governed principally by metal metalloproteinase (MMP's) and by the tissue inhibitors of MMP's (TIMP's) [22,23]. In several studies the proportion of collagen in AS can be as much as 30-60%^21^. Thus, in advanced AS, pure myocyte hypertrophy is not the only pathology that must be accounted for and consequently regress post-AVR. Were both sarcomere hypertrophy and collagen expression to be finely governed by a common pathway, coordinate regression of both would be evident [24]. However, the signaling pathway presiding over myocyte and sarcomeres appears distinct and expressed at dissimilar rates resulting in asymmetrical LVH normalization post-AVR. mRNA signaling following abrupt relief of afterload is halted within 4-6 hours in stark contrast to MMP activity which, inhibited by TIMP's, is activated late and then incompletely [25]. The resultant effect is 'accelerated" myocyte atrophy but with a more preserved interstitial composition that serves *in toto *to ameliorate the expected regression of LVH.

### Clinical Perspective

Put into perspective, the surgeon who replaces the aortic valve now has a number of explanations to account for the lack of adequate LVH regression following AVR. Even in those admirable cases in which the post-AVR gradient is reduced to < 15-20 mmHg, substantial mechanisms are operative serving to thwart the otherwise expected beneficial effects of AVR at the level of the myocardium. In short, surgical success or failure to trigger LVH regression should no longer be placed in the surgeon's prerogative.

Regarding concomitant mitral regurgitation (up to 2+; moderate) that often is associated with AS, AVR achieves improvements in MR in severe AS that are detectable by CMR and remains stable in up to 4 years of follow-up. Favorable changes appear attributable to LV and mitral valvular/annular geometry, LVH regression, less so on improved EF. Since considerable morbidity and mortality exists for simultaneous AVR and MVR, CMR suggests that AVR without MVR may be indicated in such patients.

## Conclusion

Patients with advanced AS upon surgical relief of valvular afterload, undergo rapid regression of LVH with corresponding improvements in many LV metrics measurable by CMR that is in conjunction with improvements in clinical sequelae. However, the preponderance of the surgical benefits appear early, almost truncated within the first 6 months and while durable, only minimally continue long-term out to 4 years. The long-term expected reverse remodeling appears thwarted by a myriad of so-named factors rendering incomplete the otherwise beneficial post-AVR effects. From a surgical perspective, it would seem initially apparent that any 'less then complete' normalization of LV mass after such an extended follow-up would be perceived potentially as a shortcoming of the surgical technique. From this data we can provide substantial evidence to support that this is an incorrect supposition. Whether longer-term follow-up would eventually reveal a normalized trajectory on course with historic controls is unknown but worthy of further investigation.

## Competing interests

The authors declare that they have no competing interests.

## Authors' contributions

RB conceived, designed coordinated and analyzed primary data, assisted in recruitment, IRB issues as well as wrote the manuscript. JM discussed the design of the study and performed the majority of the aortic valve replacements. SG was the nurse coordinator, recruited patients, coordinated follow-up CMR exams and all the IRB/HIPPA requirements as well as partially conceived of the secondary 4 year follow-up coordination principal study. RW performed the CMR exams and data analysis. JY performed the CMR exams and data analysis. DV statistical analysis. VR helped interpret CMR exams served as the second cardiologist on the study. GR assisted in primary data analysis and was the software engineer for the study. KC participated in the study as the cardiology fellow and separately analyzed mitral regurgitation data. MD helped to implement design, analysis and performance of the study as well as implemented optimization of the RF tissue-tagging sequence, critical discussions of the study results, critical analysis of the various drafts of the manuscript and review/approval of its' final draft. All authors read and approved the final manuscript.
